# Second Harmonic Generation microscopy reveals collagen fibres are more organised in the cervix of postmenopausal women

**DOI:** 10.1186/s12958-016-0204-7

**Published:** 2016-10-21

**Authors:** Brenda F. Narice, Nicola H. Green, Sheila MacNeil, Dilly Anumba

**Affiliations:** 1 Academic Unit of Reproductive and Developmental Medicine, University of Sheffield, Jessop Wing, Sheffield, S10 2SF UK; 2 Department of Materials Science and Engineering, Kroto Research Institute, University of Sheffield, Broad Lane, Sheffield, S3 7HQ UK

**Keywords:** Second harmonic generation microscopy, Collagen, Cervix, Menopause, Human, Alignment

## Abstract

**Background:**

During labour, the cervix undergoes a series of changes to allow the passage of the fetoplacental unit. While this visible transformation is well-described, the underlying and causative microscopic changes, in which collagen plays a major role, are poorly understood and difficult to visualise. Recent studies in mice and humans have shown that Second Harmonic Generation (SHG) microscopy, a non-destructive imaging technique, can detect changes in the cervical collagen. However, the question of whether SHG can identify changes in the arrangement of cervical collagen at different physiological stages still needs addressing. Therefore, this study aimed to compare the cervical collagen alignment between pre- and postmenopausal women using SHG and to generate proof-of-concept data prior to assessing this technique in pregnancy.

**Methods:**

Cervical biopsies from premenopausal (*n* = 4) and postmenopausal (*n* = 4) multiparous women undergoing hysterectomy for benign conditions were cross-sectionally scanned using an upright confocal microscope. SHG images were collected in Z-stacks and qualitatively evaluated using semi-quantitative scoring (0–3 in ascending degree of alignment) by assessors who were unaware of the classification of the SHG images, and quantitatively, using 2D Fourier transformation analysis. The dominant orientation and difference in dispersion of collagen fibres in each z-stack (X ± SD) was calculated and compared between groups.

**Results:**

Qualitatively, collagen fibres appeared more organised in postmenopausal women, [premenopausal: median 0, range (0–1), postmenopausal: median 1.25, range (1–3); *X* ^2^ (df = 5) = 19.35, *p* = 0.002]. Quantitatively, there was a statistically significant difference in collagen fibre dispersion between premenopausal (5.39° ± 12.68°) and postmenopausal women (−1.58° ± 8.24°), [Welch’s *t*-test (245.54) = 5.54, *p* < 0.01], with no significant differences in dispersion within each group [premenopausal, Welch’s F (7, 57.23) = 1.84, *p* = 0.098; postmenopausal, Welch’s F (7, 57.28) = 1.39, *p* = 0.23].

**Conclusion:**

These results suggest an increased alignment of cervical collagen in postmenopausal women which may result in increased stiffness and reduced compliance, confirm that SHG microscopy can provide qualitative and quantitative information about cervical collagen orientation without sample preparation, and support further research to explore SHG as a means of assessing cervical remodelling to predict the timing of term and preterm labour.

**Electronic supplementary material:**

The online version of this article (doi:10.1186/s12958-016-0204-7) contains supplementary material, which is available to authorized users.

## Background

More than 1 million babies die every year because they are born prematurely, and those who survive are at an increased risk of developmental delay, which can result in long-term morbidity that includes cerebral palsy, chronic lung dysfunction, and retinopathy of prematurity [1]. These conditions have a significant impact on the long-term wellbeing of affected children and their families. Worldwide, it is estimated that 15 million babies are born preterm every year, accounting for 12 % of deliveries in developing countries and 9 % in industrialised countries [2]. Despite many perinatal interventions, the rate of preterm birth has remained unchanged in the UK for the last 5 years while it has seen a clear rise in several other countries of the world [2, 3]. It is worth mentioning that while the US has seen a slow decline in preterm birth in the last few years, prematurity is still regarded as a serious health issue which affects approximately 380,000 births a year, i.e. 1 in 10 births in North America [4].

During pregnancy, the cervix plays several roles that include mechanical support to the uterus and gestational products, as well as immunological and endocrine functions. The cervix provides mechanical support for the growing fetoplacental unit and prevents premature delivery of the fetus through the birth canal until the time of delivery [5]. At birth, however, the rigid and closed structure of the cervix undergoes a series of extensive transformations both at a molecular and macroscopic level to allow the delivery of the fetus [6]. This process, which is known as cervical remodelling, begins early in pregnancy and involves progressive changes in the tissue microstructure which are often clinically undetectable until the last few weeks of pregnancy before labour [7]. Improved detection of these cervical remodelling changes in pregnancy may therefore facilitate the understanding of the mechanical function of the cervix as well as enable the development of new diagnostics and interventions to predict and prevent preterm birth [8].

Despite being part of the muscular uterus, the microstructure of the cervix is mainly comprised of an extracellular matrix rich in collagen I and IV, proteoglycan and hyaluronan and less than 15 % of smooth muscle, a percentage which does not seem to be age-related [5]. Collagen concentration remains stable throughout pregnancy and, therefore, other changes in the properties of collagen or the extracellular matrix (ECM) must be responsible for cervical remodelling [6, 9]. Cervical collagen fibre size and porosity progressively increase throughout pregnancy in mice and humans, and so does water concentration, which reaches its peak at the time of delivery [6, 10]. Therefore, alterations in the arrangements and cross-linking of collagen as well as the degree of hydration and elasticity are thought to be responsible for cervical remodelling [11].

Further studies on non-pregnant human cervical tissue report that levels of cervical collagen increase with age and decrease with parity, while collagen stiffness is known to increase with age but it does not seem to be altered by parity [5]. These studies highlight the importance of parity and age as confounding factors when assessing the biomechanical properties of the cervix.

With respect to the orientation of collagen fibres in the human cervix, X-ray diffraction techniques have traditionally been employed to study this feature in vitro [12]. Picro-Sirius Red, for collagen staining and subsequent microscopy, has also been used to identify and measure collagen fibre orientation in human cervical tissue and though this technique has allowed rough detection of major differences in orientation, it is time-consuming and not suitable for in vivo studies [5]. Therefore, there is still a need to develop techniques which are capable of assessing the orientation of collagen fibres in the cervix in vivo throughout pregnancy to aid our understanding of tissue physiology and pregnancy-related remodelling [13].

Most techniques that allow the study of collagen organisation are invasive, require sample preparation and ultimately damage the sample [14, 15]. Therefore, over the last 10 years, new imaging techniques such as SHG have been developed to overcome these limitations. SHG, which has become a minimally invasive tool for high-resolution imaging of non-centrosymmetric molecules such as collagen, has been well received by the biomedical community and has already proved its utility in cancer research [16]. Since the technique requires no sample preparation, collagen can be assessed without the introduction of processing artefacts and the results correlate better with in vivo events [15].

Recent studies on the use of SHG on mouse and human cervix have shown that this technique is capable of providing quantitative measurements of the morphological properties of collagen such as alignment, porosity and fibre size [6, 13, 17]. However, SHG may provide more than just descriptive information as research involving rheology suggests that some of the descriptive findings detected by SHG correlate well with the biomechanical properties of the tissue [18]. Despite these recent advances using the technique, there are still no studies on the use of SHG to quantitatively assess the alignment of collagen fibres in human cervical tissue at different physiological stages.

Therefore, the aim of the present study was to determine whether SHG is capable of observing differences in the orientation of cervical collagen fibres between pre- and postmenopausal women with similar parity. This study in conjunction with previous research shows that SHG, which is suitable for being developed into non-invasive probes for cervical monitoring, has the potential to be employed clinically to determine pre-labour cervical remodelling and its disorders [19, 20].

## Methods

### Participants and tissue collection

Non-pregnant cervical samples were obtained with informed and written consent from premenopausal (*n* = 4) and postmenopausal (*n* = 4) women undergoing laparoscopic-assisted, vaginal or total abdominal hysterectomy for benign conditions such as endometriosis, menorrhagia and prolapse. All recruited patients were multiparous (with at least 1 vaginal birth) and had normal and up-to-date cervical smear tests. Exclusion criteria included past medical history of gynaecological cancer or cervical surgery including LLETZ (Large Loop Excision of the Transformation Zone) or cone biopsy. For the premenopausal group, mean age was 39.75 ± 8.42 years, median parity was 2 and the main indication for surgery was menorrhagia. Whereas for the postmenopausal group, mean age was 64 ± 4.97 years, median parity was 2.5 and the main indication for surgical intervention was prolapse. All patients were recruited, consented and managed with approval from the South Yorkshire Research Ethics Committee (REC 08/H1310/35).

All samples were obtained in theatre by the same operator immediately after the uterus and cervix had been removed from the patient. Each biopsy involved bisection of the cervix to include the entire length and half of its diameter (4 × 2 cm on average). Each sample was then cut longitudinally along the main axis into two smaller sections: one of which was stored in phosphate buffered saline (PBS) with 0.1 mg/mL streptomycin, 0.25 μg/mL amphotericin B and 100u/mL penicillin at 4 °C while the other half was frozen at −20 °C within 20 min of it being obtained. Two different storage methods were employed to counter any artefactual changes or degradation of collagen structure arising from either of the methods.

### Second Harmonic Generation imaging

**Fig. 1:**
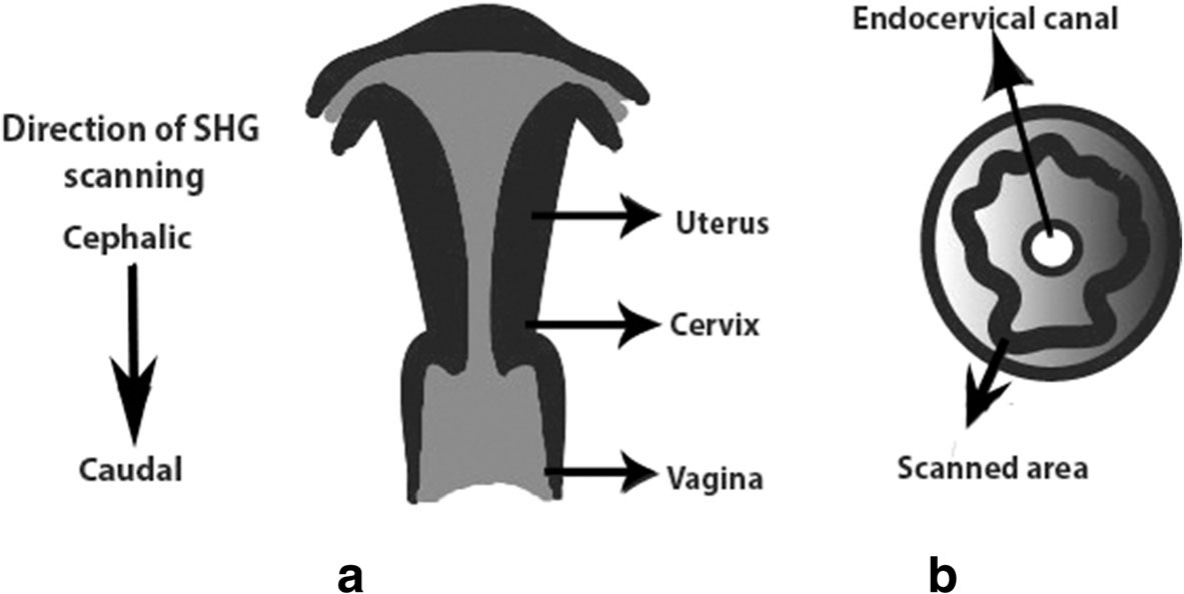
Schematic illustration of sample orientation. **a** Direction of scanning was carried out from cephalic to caudal with the cervix placed in anatomical position. **b** SHG image Z-stacks were collected from the cervical stroma adjacent to the endocervical canal as illustrated in a transverse view of the cervix

Samples preserved in PBS were scanned at room temperature immediately after being removed from the fridge while those frozen were left to defrost at room temperature for an hour before imaging. All samples were imaged using a Zeiss LSM 510 Meta upright laser-scanning confocal microscope (Oberkochen, Germany) with an Achroplan 40X/ 1.3NA oil immersion objective. A Chameleon Ti: Sapphire two-photon laser (Coherent, California, USA) tuned to 950 nm was attached to the microscope and focused onto cervical stroma resulting in a SHG signal detectable at 475 nm. In order to ensure good imaging quality of collagen fibres for orientation comparisons, the detector offset and gain were optimised for each region imaged. For consistency between samples, the cervical biopsies were scanned in anatomical position from cephalic to caudal end, and images were collected from three stromal areas: 1) the area closer to the uterine body, 2) the central area, and 3) the area closer to the vagina. Based on previous results by Reusch et al., the stromal areas examined were halfway through the endocervical canal and the lateral wall of the cervix to minimise intra-sample variability (Fig. 1) [13]. Images from each area were collected in Z-stacks of 100 images (at 1 μm intervals) but only the central 60 images of each z-stack were used in the final measurements to avoid edge effect. Since three areas were imaged in each of the 16 sections (8 PBS, 8 frozen), a total of 48 Z-stacks of 60 images each (2880 images in total) were obtained to assess the alignment of collagen in the cervical stroma.

### Two-photon excitation fluorescence

The elastin content of extirpated tissue was assessed in two premenopausal and two postmenopausal samples using two-photon excitation fluorescence (TPEF) with an excitation wavelength of 800 nm and a detection range of intrinsic fluorescence set between 447 and 597 nm [21].

### Enzymatic treatment of collagen

To verify the source of the SHG signal, some samples were treated enzymatically with collagenase A (0.120 U/ml in PBS) at 37 °C to disrupt the collagen and then rescanned 3 and 24 h later.

### Qualitative, semi-quantitative and quantitative measurements of collagen and Statistics

In order to blind the qualitative assessment of collagen alignment in the samples, ten scientists who had no prior knowledge of the project were asked to evaluate the degree of collagen alignment in 20 images using a set scoring scale ranging from 0 to 3 (0 = no alignment, 1 = some suggestion of alignment, 2 = considerable alignment and 3 = everything aligned). The assessors were blinded to menopausal status, parity and age. These semi-quantitative results were analysed statistically using the likelihood ratio test for categorical ordinal data (95 % CI).

Regarding quantitative analysis, the mean fibre direction was extracted from SHG images based on a 2D Fourier Transform analysis using Image J/ FIJI and the Directionality plug-in [22, 23]. A histogram with the peak indicative of the dominant orientation was produced for each of the Z-stacks analysed. The difference in dispersion of the collagen fibres within each Z-stack (standard deviation in the Gaussian function) was calculated and then compared within and between samples by Welch’s *t*-test and Welch’s ANOVA respectively using the statistical software SPSS v22 (IBM, 2013). Welch’s *t*-test and Welch’s ANOVA were used instead of student’s *t*-test and ANOVA due to unequal variances of the samples (significant Levene’s Test).

## Results

### Imaging of collagen with Second Harmonic Generation microscopy

**Fig. 2:**
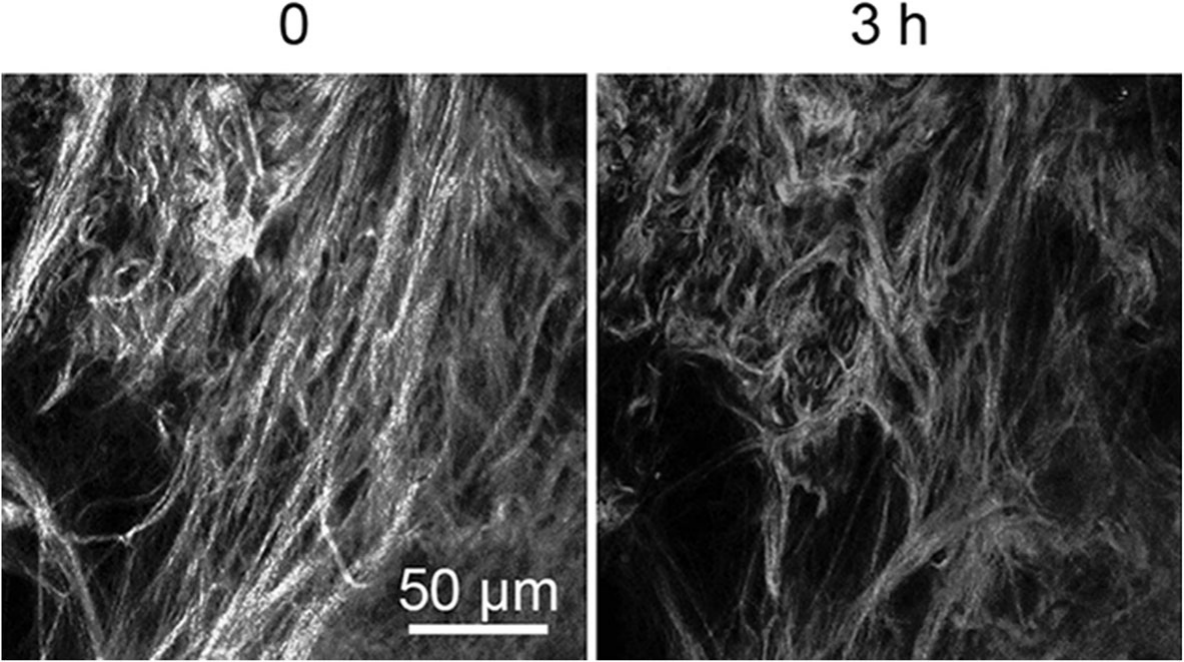
Effect of collagenase on cervical sample. PBS-preserved cervical sample scanned with SHG before and after 3-h incubation with collagenase A (same field of view)

In an attempt to explore the use of a potentially non-invasive imaging technique to assess and compare the alignment of human cervical collagen between pre- and postmenopausal women, samples were analysed with SHG microscopy. A strong signal was observed in both PBS-preserved and frozen samples when scanned with non-linear excitation. To confirm that the signal detected by SHG corresponded to collagen, three samples were incubated with collagenase A and re-imaged. Following enzymatic digestion for 3 h, samples gave SHG signals with a reduction in signal intensity relative to the untreated sample, confirming that collagen accounted for the signal detected (Fig. 2). In the four samples which were also imaged with two-photon excitation fluorescence (TPEF) to detect elastin fibres, no intrinsic fluorescence was seen.

### Qualitative and semi-quantitative changes in collagen orientation

**Fig. 3:**
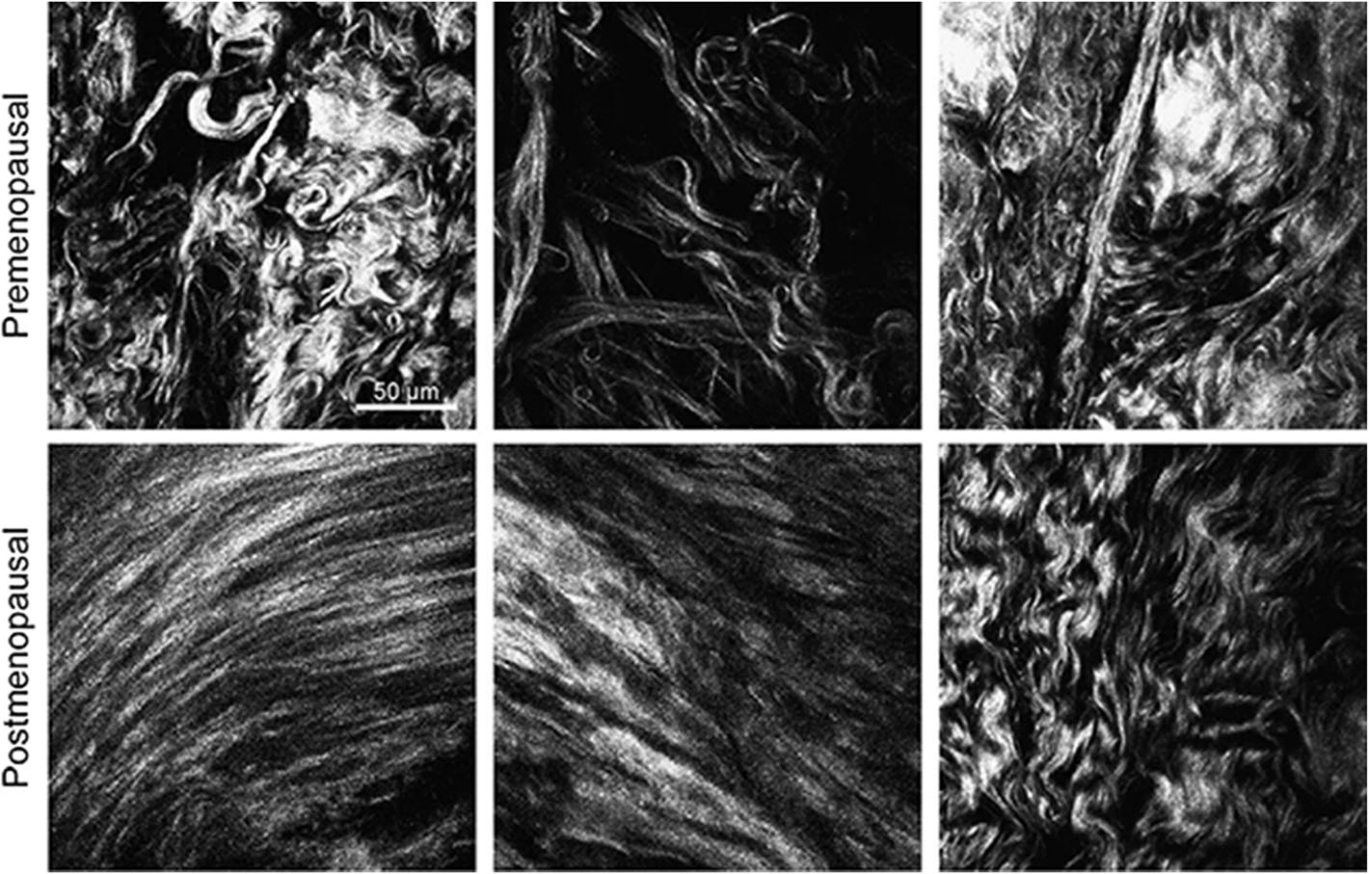
Collagen fibres as seen in the human cervix using Second Harmonic Generation. SHG signal of collagen shows less alignment of collagen fibres in premenopausal (top) than in postmenopausal samples (bottom) of cervical tissue. Cervical samples were imaged with confocal microscope, and representative images are shown. All images correspond to central areas of the cervix. Amplifier gain and offset were adjusted individually for each image to improve visualisation of collagen fibres but power excitation was kept constant

The alignment of cervical collagen fibres from the SHG images was blindly assessed by visual inspection [Additional file 1: Table S1]. The review of the images revealed clear qualitative and semi-quantitative differences in the orientation of collagen fibres between pre- [median 0, range (0–1)] and postmenopausal samples [median 1.25, range (1–3)] with a significant likelihood ratio test, *X* ^2^ (df = 5) = 19.35, *p* = 0.002. Whereas in premenopausal women there was relatively little fibre orientation with fibrillar collagen being arranged in disorganised bundles, in postmenopausal women, there was a more organised and parallel pattern with collagen fibres being co-aligned in a dominant orientation (Fig. 3).

### Quantitative changes in collagen alignment

**Fig. 4:**
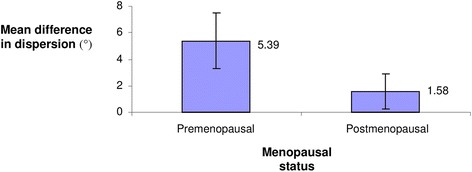
Schematic representation of the difference in the dispersion of collagen fibres based on menopausal status. Quantitative comparison of collagen alignment between premenopausal and postmenopausal cervical samples measured in degrees. The bars represent the mean difference in deviation for pre- and postmenopausal groups respectively (in absolute values) which is statistically significant with *p* < 0.01. Error bars indicate 95 % CI interval confidence for the mean difference in dispersion

The alignment of collagen fibres in each sample was quantitatively assessed using FIJI and statistically analysed with the software SPSS. To assess how many structures were arranged in the same direction, we used the FIJI Directionality plug-in based on a 2D Fourier analysis, which creates a histogram of frequency of orientations for each image. By scanning the samples in anatomical position (Fig. 1), we used the dominant orientation of collagen fibres to develop a Gaussian function, and calculated standard deviations (SDs) for the remaining fibres on a scale from −90° to 90° [Additional file 2: Table S2]. These SDs were used as a proxy for the degree of fibre alignment, with higher values describing a more disorganised pattern of collagen fibres. There was a significant difference in the dispersion of collagen fibres between pre- and postmenopausal women [Welch’s *t*-test (245. 54) = 5.54, *p* < 0.01] with a higher degree of dispersion seen in premenopausal samples (Table 1 and Fig. 4). These findings are consistent with the qualitative observations. No significant difference was found within each group [premenopausal, Welch’s F (7, 57.23) = 1.84, *p* = 0.098; postmenopausal, Welch’s F (7, 57.28) = 1.39, *p* = 0.23] or between frozen and PBS-preserved samples within pre- and postmenopausal groups [premenopausal, ANOVA F (1, 142) = 3.40, *p* = 0.067; postmenopausal, ANOVA F (1, 142) = 0.59, *p* = 0.45].

**Table 1:** Difference in the dispersion of collagen fibres

Menopausal status	Mean dispersion difference (°) 95 % CI	SD of dispersion difference (°)
Premenopausal	5.39 (3.30–7.48)	12.68
Postmenopausal	−1.58 (-2.9–-0.23)	8.24

Table 1 shows the difference in dominant orientation and dispersion of collagen fibres between cervical samples of pre- and postmenopausal women with similar parity.

## Discussion

By analysing a relatively small sample of human cervical tissue (*n* = 8), we have been able to quantify and observe a significant difference in collagen alignment between pre- and post-menopausal women after accounting for parity, with collagen fibres appearing much more aligned in postmenopausal women when compared to premenopausal. We have also been able to demonstrate that a robust collagen signal is obtained when human cervical tissues are scanned with SHG at a wavelength of 950 nm, and that the human cervix expresses little elastin as evidenced by a lack of intrinsic elastin fluorescence in all the tissues analysed, consistent with the results obtained in animal models [6].

So far the study of the orientation of collagen fibres in the cervix has been limited to invasive techniques which require extensive sample preparation such as additional staining for histology and irreversible manipulation of the sample [5]. Therefore, one of the strength of this study lies in its ability to confirm that the directionality of human cervical collagen fibres can be non-invasively detected, assessed and quantified using SHG without the need for further preparation. SHG is a relatively easy to use imaging technique capable of providing qualitative and quantitative information about collagen without destroying the sample [24]. SHG also enables single plane imaging to be reconstructed into 3D images and better penetration of tissue of up to many hundred micrometres, which facilitate understanding of in vivo processes [25]. All these characteristics make SHG an ideal technique for monitoring human tissue especially in the context of recent in vivo and in vitro studies which have shown promising results in the development of a human probe for SHG as a bedside tool to assess collagen remodelling in human cancer [16, 20]. Despite its increased relevance in the medical field in the last decade, the use of SHG to analyse cervical collagen has been limited to a few studies of porosity and fibre size in mice and only one previous study on collagen alignment in the human cervix [6, 13]. Our study, hence, addresses an original and relevant question about the use of SHG to detect and quantify changes in the morphology of collagen associated with menopause. To the best of our knowledge, there are no published studies which have formally attempted to answer this before.

However, several limitations to this study have to be considered. Firstly, given the preliminary nature of our research, the total number of samples was limited to eight, which has made controlling for other variables rather than parity and menopausal status slightly difficult. Therefore, a potential confounding factor that our current study has not addressed is the potential effect that the clinical indication for surgery could have on collagen alignment in the studied samples: most postmenopausal tissue was obtained from patients undergoing surgery for prolapse whereas younger women had other primary indications for their operations such as menorrhagia or endometriosis. However, it is worth noting that according to the Wealth’s Health Initiative Study, 41.1 % of postmenopausal women older than 60 who had not had a hysterectomy presented with some degree of pelvic organ prolapse at a routine physical examination, suggesting a strong association between prolapse and postmenopausal status and indicating that nearly half of all postmenopausal women will have some degree of prolapse [26]. We believe that replicating this study on a larger scale would help us control for more key variables and address this issue in more depth.

Secondly, even though the main aim of this pilot study was to test this methodology and obtain structural data about collagen fibres in the human cervix based on menopausal status and not information about mechanical properties, the implications of the descriptive data obtained cannot be completely elucidated without analysing its mechanical correlation. For this reason, further studies assessing the association between morphological and functional properties of human cervical collagen would be a logical next step.

Given the paucity of studies exploring the relationship between collagen morphology in the human cervix and menopausal status, there is limited data available. However, our observations seem consistent with current evidence that cervical collagen fibres behave like a rope: when collagen fibres are pulled along the axis of its triple helix, as appears to be the case in pelvic organ prolapse, they straighten out, whereas when compressed, they buckle [27].

Our observations suggest the existence of some degree of correlation between the alignment of cervical collagen fibres and the tensile response and compliance of the tissue. We hypothesise that a lower degree of cross-linking and alignment in cervical collagen, as seen in premenopausal women, may result in reduced stiffness and increased compliance which are necessary mechanical properties for cervical remodelling in pregnancy. Understandably, the ability of the cervix to efface and dilate in order to enable the birth process is no longer needed in postmenopausal women, and this seems to be reflected in a more organised but less compliant cervical tissue after menopause. Our interpretation would be in keeping with studies carried out by Yoshida et al. who reported that cervical collagen needs to be more disorganised and less cross-linked to allow dilatation during birth [28]. It would also seem to support the work by Gill et al. who found greater stromal autofluorescence attributed to cross-linking molecules in the cervical collagen of postmenopausal women compared to their premenopausal counterparts [29].

Similarly, some authors have studied the impact of menopause on human tendons and fascias which, like the cervix, are also mainly comprised of collagen. The study of the arcus tendineous fasciae pelvis (ATFP), a structure which gives support to the anterior vagina, in both pre- and postmenopausal women, for example, showed that the changes in collagen I, rather than in elastin or in smooth muscle, appear to compromise the tensile strength of the tissue after menopause [30]. Further analysis of tendons in women exposed to low estradiol levels has shown a higher proportion of immature cross-links, relatively higher compliance and lower stiffness when compared to postmenopausal women with no hormonal replacement which supports the hypothesis that oestrogen levels in the bloodstream influence the biomechanical properties of collagen [31, 32].

Finally, our study, with its strengths and limitations, highlights the existing gap in knowledge about the morphology and behaviour of cervical collagen in women and emphasises the need for further and larger studies to explore how collagen alignment is affected by menopause, pregnancy and problems of the pelvic floor such as extensive prolapse of the pelvic organs.

## Conclusions

SHG microscopy can be used to visualise collagen and assess the orientation of collagen fibres in the human cervix. Differences in cervical collagen orientation between pre- and postmenopausal women can be easily observed and quantified with SHG using user-friendly imaging tools. This technique suggests that postmenopausal women have an increased alignment of collagen fibres in the cervix compared to their premenopausal counterparts which would result in increased stiffness and reduced compliance of the tissue.

The orientation of collagen fibres is reported to play an essential role during the cervical remodelling process both in normal and preterm labour [11].

Accordingly, developing and perfecting an imaging technique which has the potential to non-destructively monitor and quantify changes in cervical collagen alignment in vivo would be very valuable. Ultimately, we would like to employ this technique to assess cervical remodelling prior to human birth.

## Additional files

Additional file 1: Table S1. Raw data from qualitative assessment of SHG images. (XLS 18 kb)
Additional file 2: Table S2. Raw data from quantitative assessment of SHG images using 2D Fourier analysis. (XLS 61 kb)
